# Insufficient Nutrition and Mortality Risk in Septic Patients Admitted to ICU with a Focus on Immune Dysfunction

**DOI:** 10.3390/nu11020367

**Published:** 2019-02-10

**Authors:** Kai-Yin Hung, Yu-Mu Chen, Chin-Chou Wang, Yi-Hsi Wang, Chiung-Yu Lin, Ya-Ting Chang, Kuo-Tung Huang, Meng-Chih Lin, Wen-Feng Fang

**Affiliations:** 1 Division of Pulmonary and Critical Care Medicine, Department of Internal Medicine, Kaohsiung Chang Gung Memorial Hospital, Chang Gung University College of Medicine, Niaosung, Kaohsiung 833, Taiwan; redrosahung@yahoo.com.tw (K.-Y.H.); blackie@adm.cgmh.org.tw (Y.-M.C.); ccwang5202@yahoo.com.tw (C.-C.W.); yihsi@cgmh.org.tw (Y.-H.W.); chiungyu@cgmh.org.tw (C.-Y.L.); chphyllis@yahoo.com.tw (Y.-T.C.); jelly@cgmh.org.tw (K.-T.H.); mengchih@cgmh.org.tw (M.-C.L.); 2 Department of nutritional Therapy, Kaohsiung Chang Gung Memorial Hospital, Kaohsiung 833, Taiwan; 3 Graduate Institute of Clinical Medical Sciences, College of Medicine, Chang Gung University, Kaohsiung Chang Gung Memorial Hospital, Kaohsiung 833, Taiwan; 4 Department of Respiratory Care, Chang Gung University of Science and Technology, Chiayi 813, Taiwan

**Keywords:** sepsis, ICU, HLA-DR, nutrition

## Abstract

Immune dysfunction is seen both in sepsis patients and in those with malnutrition. This study aimed to determine whether insufficient nutrition and immune dysfunction have a synergistic effect on mortality in critically ill septic patients. We conducted a prospective observational study from adult sepsis patients admitted to intensive care units (ICUs) between August 2013 and June 2016. Baseline characteristics including age, gender, body mass index, NUTRIC, Acute Physiology and Chronic Health Evaluation (APACHE) II and Sequential Organ Failure Assessment (SOFA) scores were recorded. Immune dysfunction, defined by human leukocyte antigen DR (HLA-DR) expression, was tested at days 1, 3, and 7 of ICU admission. The study included 151 patients with sepsis who were admitted to the ICU. The 28-day survivors had higher day 7 caloric intakes (89% vs. 73%, *p* = 0.042) and higher day 1-HLA-DR expression (88.4 vs. 79.1, *p* = 0.045). The cut-off points of day 7 caloric intake and day 1-HLA-DR determined by operating characteristic curves were 65.1% and 87.2%, respectively. Immune dysfunction was defined as patients with day 1-HLA-DR < 87.2%. Insufficient nutrition had no influence on survival outcomes in patients with immune dysfunction. However, patients with insufficient nutrition had poor prognosis when they were immune competent. Insufficient nutrition and immune dysfunction did not have a synergistic effect on mortality in critically ill septic patients.

## 1. Introduction

The association between caloric delivery and clinical outcomes in critically ill patients is a controversial topic in the present day [1]. In past studies, it was shown that early parenteral nutrition supplementation in critically ill patients who could not reach a caloric target failed to improve survival outcomes [2,3,4,5]. Several observational studies have demonstrated that insufficient energy delivery is associated with higher mortality rates [6,7]. A meta-analysis conducted by Marik and Hooper found that intentional hypocaloric feeding was not associated with risk of acquired infections or hospital mortality compared to normocaloric nutrition [8]. A prospective pilot study conducted by Petros et al. found that hypocaloric feeding was associated with more nosocomial infections in critically ill patients [9]. A recent prospective randomized study conducted by Arabi et al. revealed that permissive underfeeding was not associated with higher mortality rates in critically ill patients [10]. Another study conducted by Chapman et al. found that energy-dense enteral nutrition had no survival benefit compared to enteral nutrition, in critically ill patients [11]. Amino acid infusion could not prevent muscle wasting in intensive care unit (ICU) patients [12,13]. Both above studies comprised less than 50% sepsis patients. However, patients with sepsis have worse clinical outcomes and might have distinct immune profiles compared to those without [14,15,16,17,18]. 

Sepsis, a life-threatening organs dysfunction, mostly caused by bacterial infection, is associated with dysregulated host responses to infection [19]. Despite decreasing mortality rates of sepsis and septic shock, the incidence is increasing [20]. The immune dysregulation induced by sepsis promotes endothelial dysfunction and alters hemostasis and microcirculation [21]. Based on the above reasons, we conducted a study consisting purely of septic patients to reduce confounding. Immune dysfunction was seen both in sepsis patients and in people with malnutrition [22,23,24]. We hypothesized that insufficient nutrition and immune dysfunction may have a synergistic effect on mortality in critically ill septic patients. 

## 2. Materials and Methods 

### 2.1. Patient Population

We conducted a prospective observational study from August 2013 to May 2016. We included patients with sepsis who were admitted to medical intensive care units (ICUs). All patients admitted to participating units were screened for eligibility. Patients were excluded if they met one of the following criteria: (1) Those who were <18 years of age; (2) those who had ICU waiting times longer than 24 h after diagnosis of sepsis; (3) those who received granulocyte-colony stimulating factor 1 week prior to ICU admission; or (4) those who were contraindicated to receive enteral feeding, including hollow organ perforation, gastrointestinal obstruction, gastrointestinal bleeding, ileus, etc.

All patients received blood sampling at days 1, 3 and 7 of ICU admission. Patients received enteral feeding as soon as possible if there was no contraindication. The recommended daily amount of energy the patients required was evaluated by registered dietitians in our ICU. The caloric meet per day was calculated by dividing their actual daily intake of calories by their recommended daily amount of calories. Clinical parameters including age, sex, body mass index (BMI), Acute Physiology and Chronic Health Evaluation (APACHE) II score, Sequential Organ Failure Assessment (SOFA) score, NUTRIC score [25] (including six parameters: Age, APACHE II, SOFA, number of comorbidities, IL-6 and days in hospital before admission to ICU) and daily caloric delivery were recorded. All patients were observed until death or until discharged from the hospital. The primary outcome was 28-day mortality (day 1 was defined as ICU admission day). The study was approved by the Institutional Review Board with informed consent obtained from patients or their surrogates. The trial was registered on ClinicalTrials.gov, ID: NCT02887274

### 2.2. Definitions

The definition of severe sepsis was first adapted from the 2001 International Sepsis Definitions Conference and the Surviving Sepsis Campaign [26]. All enrolled patients fulfilled the definition of sepsis from the Third International Consensus Definitions for Sepsis and Septic Shock (Sepsis-3) [27]. We then adapted the new definition. Day 1 was defined as ICU admission day. Body mass index (BMI) was defined as dividing weight in kilograms by the square of height in meters. Plasma and peripheral blood mononuclear cell (PBMC) preparations, Monocyte HLA-DR expression measurements by flow cytometry [28], and Milliplex assays were mentioned in our previous studies [29,30].

### 2.3. Statistical Analyses 

Statistical analyses were performed using MedCalc (version 14.10.2, Software, Ostend, Belgium). Categorical variables were compared using the chi-square test or Fisher’s exact test where appropriate, and continuous variables were analyzed using Student’s t-test or the Mann–Whitney U test where appropriate. Collinearity of similar variables including the NUTRIC score, APACHE II and SOFA scores was tested using Scatter plot, correlation analysis and variance Inflation Factor. A logistic regression analysis model was used for multivariate analyses of the effect of the prognostic factors on patient survival. A receiver operating characteristic (ROC) curve and Youden’s index were used to determine the best cut-off values for 28-day mortality that were statistically significant in the univariable analysis. The Kaplan–Meier method and the log-rank test were used to determine the effect of the prognostic factors on patient survival. The pair-wise deletion method was applied for missing values. A *p*-value of <0.05 was considered statistically significant.

## 3. Results

### 3.1. Patient Characteristics between 28-Day Survivors and Non-Survivors.

**Figure 1:**
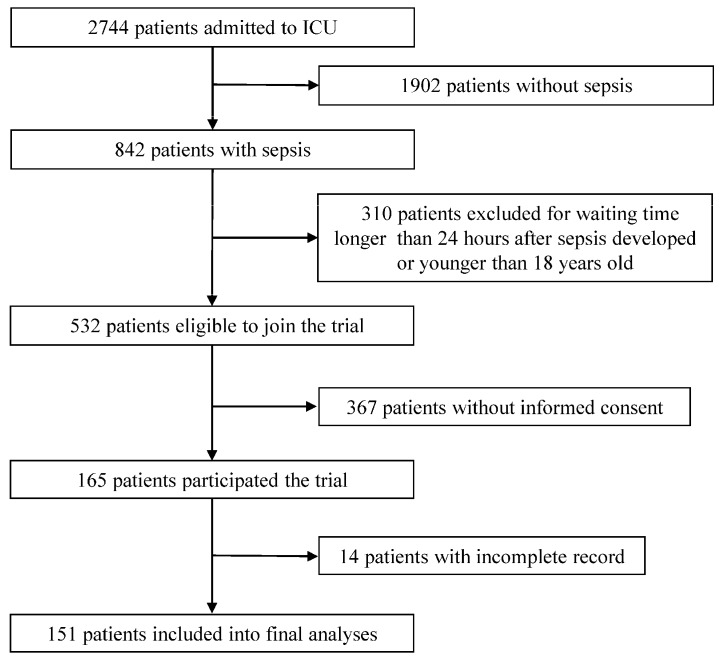
Patient recruitment and assignment.

Of the 2744 patients admitted to the ICU from August 2013 to June 2016, 151 sepsis patients were included in the final analyses (Figure 1).

Patients surviving at 28 days after ICU admission had lower SOFA scores than non-survivors (8.91 vs. 11.90, *p* < 0.001), lower NUTRIC scores (5.52 vs. 6.30, *p* = 0.023), higher day 7 caloric meets (89% vs. 73%, *p* = 0.042), and higher day 1-HLA-DR expression (88.37% vs. 79.13%, *p* = 0.045) (Table 1). The clinical characteristics taken into account are: age, sex, BMI, focus of infection, history of CAD, HTN, DM, stroke, CKD, cancer cirrhosis, IL-6, caloric meet on day 1 and day 3, and HLA-DR expression on day 3 and day 7; the amount of protein intake was not significantly associated with 28-day mortality.

### 3.2. Risk Factors of Lower Day 7 Caloric Meet

Patients with a first quartile of day 7 caloric meet were more likely to be female (*p* = 0.011), have chronic kidney disease (*p* = 0.041) and have a history of cancer (*p* = 0.021) (Table 2).

### 3.3. Impact of Immune Dysfunction and Day 7 Caloric Meet on Survival Outcome

**Figure 2:**
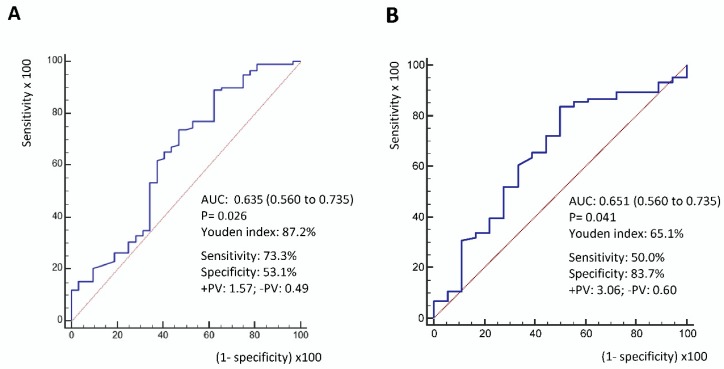
(**A**) The ROC curve of day 1-HLA-DR for 28-day mortality prediction. The best cut-off point for day 1-HLA-DR determined using the ROC curve and Youden index was 87.2%. (**B**) The ROC curve of day 7 caloric meet for 28-day mortality prediction. The best cut-off point for day 7 caloric meets determined using the ROC curve and Youden index was 65.1%. PV: predictive value.

The best cut-off point for day 1-HLA-DR, as determined using the ROC curve and Youden index, was 87.2% (Figure 2A). Immune dysfunction was defined as patients with a day 1-HLA-DR < 87.2%, and patients with day 1-HLA-DR ≥ 87.2% were viewed as immune competent. Forty-seven (31.3%) patients had immune dysfunction. Patients with immune dysfunction were more likely to be older (71.0 vs. 65.6 years, *p* = 0.042) and have a lower BMI (21.7 vs. 24.0%, *p* = 0.007) (Table S1). The best cut-off point for day 7 caloric meet, as determined using the ROC curve and Youden index was 65.1% (Figure 2B). Patients were divided into high and low day 7 caloric meets, based on the above cut-off point. Twenty-five (20.5%) patients had a low day 7 caloric meet. 

**Figure 3:**
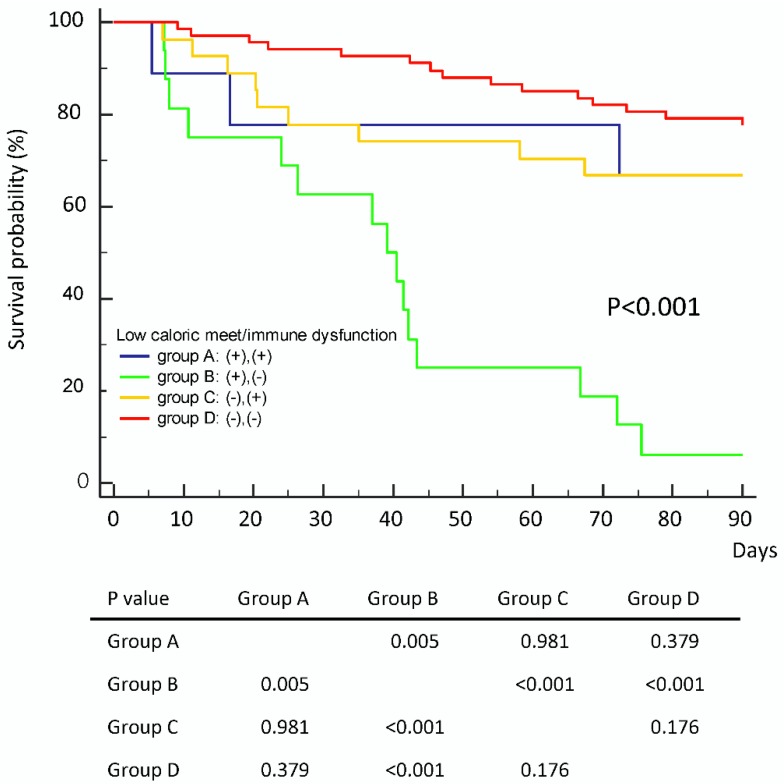
Impact of day 7 caloric meet on survival outcome in patients with or without immune dysfunction. Patients with higher day 7 caloric meet and normal immune status had the best prognosis. Patients with lower day 7 caloric meet and normal immune status had a worse prognosis.

Patients were divided into four subgroups based on day 7 caloric meet and immune dysfunction status (Figure 3). Group A (*n* = 9): Patients with both low day 7 caloric meet and immune dysfunction; Group B (*n* = 16): Patients with low day 7 caloric meet, without immune dysfunction; Group C (*n* = 28): Patients with high day 7 caloric meet and immune dysfunction; Group D (*n* = 69): patients with high day 7 caloric meet, without immune dysfunction. Baseline clinical characteristics between the four groups of patients are shown in Table S2. Group D patients had the best prognosis (*p* = 0.379, *p* < 0.001, *p* = 0.176 when group D was compared to groups A, B, and C respectively). Group B patients had a worse prognosis (*p* = 0.005, *p* < 0.001, *p* < 0.001 when group B was compared to groups A, C and D respectively) (Figure 3). In the subgroup of patients with immune dysfunction, insufficient nutrition had no influence on survival outcomes (*p* = 0.981). Surprisingly, insufficient nutrition patients had a poor prognosis only when they were immune competent (*p* < 0.001).

### 3.4. Impact of NUTRIC Score and Day 7 Caloric Meet on Survival Outcome 

**Figure 4:**
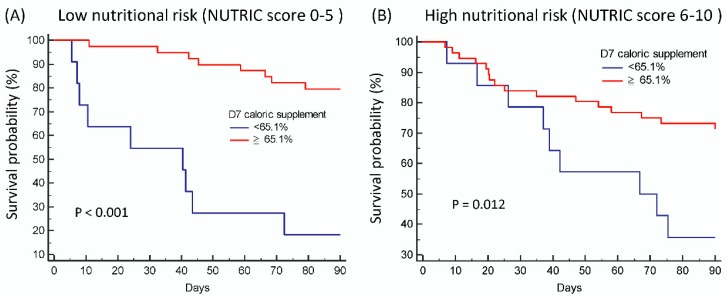
Impact of day 7 caloric meet on survival outcome in patients with high or low NUTRIC score. Patients with a lower day 7 caloric meet had a poor prognosis both in those with (**A**) low nutritional risk (NUTRIC score 0–5) and (**B**) high nutritional risk (NUTRIC score 6–10).

Patients were divided into high nutritional risk if they had a NUTRIC score of 6–10 and low nutrition risk if they had a NUTRIC score of 0–5. Of the 122 patients available for NUTRIC scoring, 71 (58.2%) patients were categorized as high nutritional risk and 51 (41.8%) patients as low nutritional risk. Patients with a lower day 7 caloric meet had poor prognosis, both in those with low (Figure 4A) and high (Figure 4B) nutritional risk (*p* < 0.001 and *p* = 0.012 respectively).

### 3.5. Multivariate Analysis of Clinical Characteristics and Immune Status and Their Impact on Mortality

Factors that significantly affected a patient’s survival in univariate analysis were baseline SOFA score, day 1 HLA-DR, day 7 caloric meet, and NUTRIC score (Table 1). The SOFA score was not included in multivariate analysis for its collinearity with the NUTRIC score. (Figure S1.) Day 1 HLA-DR, day 7 caloric meet and NUTRIC score were subsequently included in the multivariate analysis. In the multivariate analysis, day 7 caloric meet was significantly associated with 28-day mortality (*p* = 0.022) (Table 3)

## 4. Discussion

Our study found that immune-competent patients were more likely to be influenced by insufficient nutrition. On the contrary, insufficient nutrition did not influence survival outcomes in patients with immune dysfunction. This phenomenon has been seldom mentioned before and the mechanism behind this phenomenon is not clear. Several possible mechanisms may lead to these outcomes. First, caloric restriction promotes mammalian cell survival through its effects on regulating inflammatory pathways in previous studies [31]. We speculated that caloric restriction in the acute phase of infection may alleviate inflammation reaction and its collateral damage by regulating metabolic, hormonal, and inflammatory pathways. Second, caloric restriction was associated with better blood glucose control, as it decreased the incidence of hyperglycemia [31,32]. Short-term hyperglycemia could impair innate immune responses to infection [33]. In patients who already had immune dysfunction, poor blood glucose control exacerbated immune dysfunction status. These hypotheses can at least partly explain why immune dysfunction patients were less likely to be harmed by insufficient nutrition.

Hospitalized patients are more likely to suffer from malnutrition, which may be due to a disease-related catabolic state or inadequate nutrition supply [34,35,36,37]. The risk of malnutrition in the ICU ranged from 6–42% based on the adopted definition and the study patient population [35,38,39,40]. Patients with several underlying diseases were mentioned to be at risk of malnutrition including inflammatory bowel diseases, heart failure, lung diseases [40], and cancer [41]. Malnutrition impairs cellular immunity, which increases ICU mortality and prolonged hospital stays [42,43,44,45]. Previous studies revealed that some patients dying of sepsis have marked immunosuppression [15,16]. Lower monocyte HLA-DR expression is a marker of immune paralysis [28], and patients with lower monocyte HLA-DR expression were at higher risk of bacterial infection [46]. Our study revealed that patients surviving 28 days after ICU admission had less immunosuppression which was evidenced by higher day 1 HLA-DR expression. 

A previous study revealed that insufficient energy delivery is associated with higher blood stream infection rates [6], increased length of days with mechanical ventilation [42], and higher mortality rates [43]. Early enteral feeding was proven to reduce ICU and hospital mortality and has become the current daily practice guideline [44]. On the other hand, early parenteral nutrition for nutrition augmentation was abandoned because there were increased complications and no improvement in mortality rates [5]. Our study revealed that female patients with chronic kidney disease and having a cancer history were more likely to have insufficient nutrition. Consistent with previous studies, our study revealed that patients with insufficient nutrition had a higher mortality rate.

Our study had several limitations. First, correlation does not mean causality and insufficient enteral nutrition may be caused by higher severity of illness rather than being a cause of higher mortality. Further prospective controlled and randomized studies are required to illuminate the cause and effect between insufficient enteral nutrition, disease severity and mortality rates. Second, potential inaccuracies in weight measurements were a non-negligible issue in ICU, and serial body weight changes were mostly missing due to difficulty in measuring the weight. Third, the lack of indirect calorimetry made accurate determinations of energy expenditure impossible. Finally, data regarding blood transfusions received and the type of antibiotics or steroids prescribed were largely missing, which could potentially influence immune function as well as target calorie needs. 

## 5. Conclusions

Insufficient nutrition and immune dysfunction did not have a synergistic effect on mortality in critically ill septic patients. 

## Figures and Tables

**Table 1:** Clinical characteristics and immune status between 28-day survivors and non-survivors. (*n* = 151) *.

	All (*n* = 151)	Non-Survivor (*n* = 30)	Survivor (*n* = 121)	*p*
Age (years), mean (SD)	67.3 (15.1)	67.1 (13.5)	67.4 (15.5)	0.758
Male, n (%)	60 (39.7)	13 (43.3)	47 (38.8)	0.653
Body Mass Index, mean (SD)	23.2 (5.0)	24.6 (4.6)	22.8 (5.0)	0.056
Coronary artery disease, n (%)	45 (29.8)	8 (26.7)	37 (30.6)	0.675
Hypertension, n (%)	87 (57.6)	18 (60.0)	69 (57.0)	0.768
Diabetes mellitus, n (%)	75 (49.7)	16 (53.3)	59 (48.8)	0.654
Stroke, n (%)	32 (21.2)	6 (20.0)	26 (21.5)	0.858
Chronic kidney disease, n (%)	34 (22.5)	9 (30.0)	25 (20.7)	0.273
Infection focus				0.068
Lung	95 (62.9)	16 (16.8)	79 (83.2)	
Urinary tract	20 (13.2)	2 (10.0)	18 (90.0)	
Intra-abdomen	6 (4.0)	1 (16.7)	5 (83.3)	
Others	30 (19.9)	11 (36.7)	19 (63.3)	
Hemodialysis, n (%)	30 (19.9)	8 (26.7)	22 (73.3)	0.297
Cancer, n (%)	29 (19.2)	7 (23.3)	22 (18.2)	0.209
Cirrhosis, n (%)	6 (4)	3 (10)	3 (2.5)	0.059
SOFA score, mean (SD)	9.5 (3.6)	11.9 (4.2)	8.9 (3.2)	<0.001
APAHCE-II, mean (SD)	26.01 (8.77)	27.57 (7.77)	25.62 (8.99)	0.240
Interleukin-6, pg/mL, mean (SD)	313.2 (1351.4)	134.1 (293.7)	1035.8 (2901.0)	0.100
NUTRIC score, mean (SD)	5.7 (1.9)	6.3 (2.0)	5.5 (1.8)	0.023
Day 1 caloric intake, mean (SD)	617.7 (439.8)	490.2 (444.6)	649.1 (434.8)	0.081
Day 1 caloric meet, mean (SD)	0.43 (0.30)	0.34 (0.31)	0.45 (0.30)	0.069
Day 1 protein intake, g, mean (SD)	18.6 (17.7)	13.5 (15.8)	19.8 (18.0)	0.089
Day 1 HLA-DR (%), mean (SD)	86.5 (15.8)	79.1 (21.5)	88.4 (13.5)	0.045
Day 3 caloric intake, mean (SD) **	1053.4 (496.1)	882.0 (573.0)	1087.1 (475.0)	0.070
Day 3 caloric meet, mean (SD)	0.74 (0.36)	0.62 (0.42)	0.76 (0.34)	0.072
Day 3 protein intake, g, mean (SD)	36.9 (22.4)	37.9 (21.9)	36.5 (22.7)	0.737
Day 3 HLA-DR (%), mean (SD)	89.8 (12.2)	85.1 (15.4)	90.6 (11.4)	0.052
Day 7 caloric intake, mean (SD) ***	869.8 (304.7)	730.4 (310.3)	890.8 (299.7)	0.049
Day 7 caloric meet, mean (SD)	0.87 (0.30)	0.73 (0.31)	0.89 (0.30)	0.042
Day 7 protein intake, g, mean (SD)	48.3 (20.7)	43.8 (21.3)	50.3 (20.3)	0.114
Day 7 HLA-DR (%), mean (SD)	93.6 (9.9)	83.9 (21.1)	94.7 (7.1)	0.085

* Data were measured at the first day of ICU admission unless otherwise mentioned; ** 140 patients who survived at day 3 were analyzed for day 3 caloric meet and day 3 HLA-DR; *** 122 patients who survived at day 7 were analyzed for day 7 caloric meet and day 7 HLA-DR; Abbreviations: SOFA: Sequential Organ Failure Assessment; HLA-DR: Human Leukocyte Antigen DR.

**Table 2:** Clinical characteristics and immune status between patients within different quartiles of caloric meet (*n* = 122).

Day 7 Caloric Meet	First Quartile (<71.0 %, *n* = 30)	Second Quartile (71.0%–93.8%, *n* = 31)	Third Quartile (93.8%–107.4%, *n* = 31)	Fourth Quartile (>107.4%, *n* = 30)	*p*
Age (years), mean (SD)	69.6 (15.6)	68.2 (15.5)	69.7 (12.8)	65.7 (16.6)	0.710
Male, n (%)	11 (36.7)	7 (22.6)	11 (35.5)	19 (63.3)	0.011
Body Mass Index, mean (SD)	22.8 (4.7)	23.0 (6.8)	22.0 (4.7)	23.8 (4.1)	0.598
Coronary artery disease, n (%)	9 (30.0)	4 (12.9)	9 (29.0)	13 (43.3)	0.074
Hypertension, n (%)	14 (46.7)	20 (64.5)	18 (58.1)	17 (56.7)	0.568
Diabetes mellitus, n (%)	13 (43.3)	12 (38.7)	15 (48.4)	18 (60.0)	0.383
Stroke, n (%)	8 (26.7)	8 (26.7)	5 (16.1)	4 (13.3)	0.241
Chronic kidney disease, n (%)	11 (36.7)	8 (25.8)	3 (9.7)	4 (13.3)	0.041
Cancer, n (%)	12 (40.0)	5 (16.1)	5 (16.1)	3 (10.0)	0.021
Cirrhosis, n (%)	3 (10.0)	0 (0.0)	1 (3.2)	1 (3.3)	0.252
SOFA score, mean (SD)	10.7 (4.6)	8.9 (3.2)	9.0 (3.0)	8.7 (2.8)	0.105
Interleukin-6, µ/mL, mean (SD)	198.2 (326.0)	163.2 (327.2)	94.3 (159.8)	56.2 (107.3)	0.118
NUTRIC score, mean (SD)	5.7 (2.0)	5.6 (1.9)	5.7 (1.8)	5.3 (1.8)	0.783
HLA-DR (%), mean (SD)	81.9 (17.8)	87.4 (13.8)	90.4 (13.0)	89.1 (13.4)	0.122

Abbreviations: SOFA: Sequential Organ Failure Assessment; HLA-DR: Human Leukocyte Antigen DR.

**Table 3:** Multivariate analysis of clinical characteristics and immune status for 28-day mortality prediction. (*n* = 122).

	β	S.E.	Wals	df	sig	Exp(β) (95% C.I.)
NUTRIC score	0.004	0.147	0.001	1	0.976	1.004 (0.753–1.339)
Day 1 HLA-DR (≥87.2% vs. <87.2%)	0.544	0.574	0.898	1	0.343	1.723 (0.559–5.307)
day 7 caloric meet (≥65.1% vs. <65.1%)	1.280	0.560	5.220	1	0.022	3.596 (1.200–10.782)

Abbreviations: S.E.: standard error; Wals: rating scale; df: degrees of freedom; sig: statistical significance; Exp(β): odds ratio; C.I.: confidence interval.

## Supplementary Materials

The following are available online at https://www.mdpi.com/2072-6643/11/2/367/s1, Table S1: Clinical characteristics and immune status between patients with or without immune dysfunction (Day 1-HLA-DR <87.2%) (*n* = 151); Table S2: Clinical characteristics in patients with different day 7 caloric meet and day 1 HLA-DR (*n* = 122); Figure S1: Scatter plot of NUTRIC score (X) and SOFA score.
