# Supplementary material for: Insufficient Nutrition and Mortality Risk in Septic Patients Admitted to ICU with a Focus on Immune Dysfunction
Source: Nutrients. 2019 Feb 10;11(2):367. doi: 10.3390/nu11020367 (PMC6412372; doi:10.3390/nu11020367)
Supplement: Supplementary file 1 [file nutrients-11-00367-s001.pdf]

**Supplement table 1. Clinical characteristics and immune status between patients with or without immune dysfunction (Day 1-HLA-DR <87.2%) (n = 151).**

| | Day 1-HLA-DR <87.2% (n=48, 31.8%) | Day 1-HLA-DR ≥87.2% (n=103, 68.2%) | p |
|---|---|---|---|
| Age (years), mean (SD) | 71.0 (13.6) | 65.6 (15.6) | 0.042 |
| Male, n (%) | 23 (47.9) | 37 (35.9) | 0.161 |
| Body Mass Index, mean (SD) | 21.5 (5.0) | 24.0 (4.8) | 0.007 |
| Coronary artery disease, n (%) | 13 (27.1) | 32 (31.1) | 0.618 |
| Hypertension, n (%) | 26 (54.2) | 61 (59.2) | 0.558 |
| Diabetes mellitus, n (%) | 22 (45.8) | 53 (51.5) | 0.520 |
| Stroke, n (%) | 11(22.9) | 21(20.4) | 0.723 |
| Chronic kidney disease, n (%) | 11 (22.9) | 23 (22.3) | 0.936 |
| Cancer, n (%) | 9 (18.8) | 20 (19.4) | 0.923 |
| Cirrhosis, n (%) | 2 (4.2) | 4 (3.9) | 0.934 |
| SOFA score, mean (SD) | 9.6 (3.4) | 9.4 (3.7) | 0.791 |
| Interleukin-6, pg/mL, mean (SD) | 312.2 (1132.3) | 313.7 (1147.4) | 0.960 |
| NUTRIC score, mean (SD) | 6.1 (1.8) | 5.5 (1.9) | 0.075 |

Abbreviations: SOFA: Sequential Organ Failure Assessment; HLA-DR: Human Leukocyte Antigen DR.

**Supplement table 2. Clinical characteristics in patients with different day 7 caloric meet and day 1 HLA-DR (n = 122).**

| Day 7 caloric meet and day 1 HLA-DR | Group A: low day 7 caloric meet, with immune dysfunction (n=9) | Group B: low day 7 caloric meet, without immune dysfunction (n=16) | Group C: high day 7 caloric meet, with immune dysfunction (n=28) | Group D: high day 7 caloric meet, without immune dysfunction (n=69) | p |
|---|---|---|---|---|---|
| Age (years), mean (SD) | 76.7 (15.0) | 63.3(15.3) | 70.5(13.8) | 65.3(15.3) | 0.143 |
| Male, n (%) | 3(33.3) | 6(37.5) | 14(50.0) | 25(36.2) | 0.621 |
| Body Mass Index, mean (SD) | 18.9(2.9) | 24.3(4.3) | 21.7(5.5) | 23.7(5.0) | 0.019 |
| Coronary artery disease, n (%) | 2 (22.2) | 5 (31.3) | 6 (21.4) | 22 (31.9) | 0.729 |
| Hypertension, n (%) | 4(44.4) | 7(43.8) | 15(53.6) | 43(62.3) | 0.451 |
| Diabetes mellitus, n (%) | 3(33.3) | 8(50.0) | 14(50.0) | 34(49.3) | 0.829 |
| Stroke, n (%) | 2(22.2) | 3(18.8) | 7(25.0) | 15(21.7) | 0.970 |
| Chronic kidney disease, n (%) | 3(33.3) | 6(37.5) | 5(17.9) | 12(17.4) | 0.250 |
| Cancer, n (%) | 4 (44.4) | 7(43.8) | 3(10.7) | 11(15.9) | 0.012 |
| Cirrhosis, n (%) | 1(11.1) | 2(12.5) | 0(0) | 2(2.9) | 0.142 |
| SOFA score, mean (SD) | 9.6 (4.2) | 11.7 (5.2) | 9.1 (2.9) | 8.7 (3.0) | 0.025 |
| NUTRIC score, mean (SD) | 6.1 (2.2) | 5.4 (2.2) | 5.9 (1.8) | 5.4 (1.9) | 0.493 |

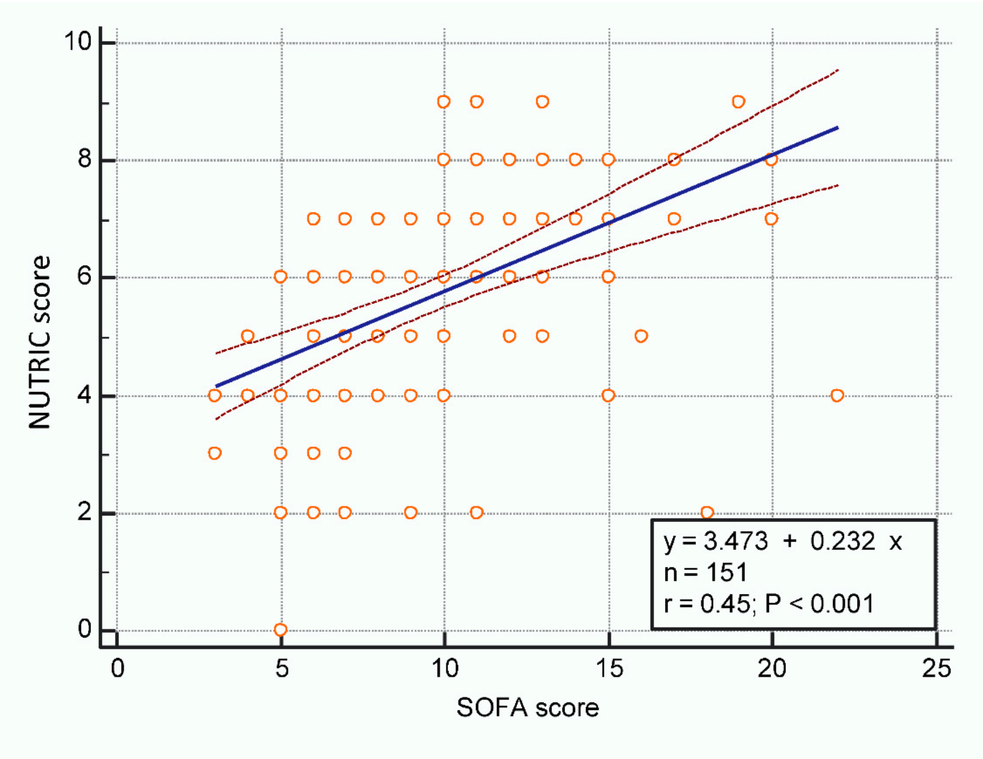


**Figure S1.** Scatter plot of NUTRIC score (X) and SOFA score.
